# Successful topical treatment of a gunshot wound: A case report

**DOI:** 10.1016/j.tcr.2023.100787

**Published:** 2023-02-07

**Authors:** Saleheh Tajalli, Mazlome Hamzekhani, Faezeh Farzi, Mohsen Saeidi Abu-Es'haghi, Asad Imani

**Affiliations:** a Nursing and Midwifery Care Research Center, School of Nursing and Midwifery, Iran University of Medical Sciences, Tehran, Iran; b Department of Midwifery, School of Nursing and Midwifery, Shahroud University of Medical Sciences, Iran; c Imam Khomeini Hospital, Ilam University of Medical Sciences, Ilam, Iran; d Lordegan Shohada Hospital, Shahrekord, Iran; e Department of Nursing, Faculty of Nursing and Midwifery, Ilam University of Medical Sciences, Ilam, Iran. Student Research Committee, Faculty of Nursing and Midwifery, Ahvaz Jundishapur University of Medical Sciences, Ahvaz, Iran

**Keywords:** Gunshot, Wound, Bandages, Nurse practitioners, Wound healing

## Abstract

Gunshot wound leads to deep soft tissue damages known as penetrating trauma. Wound healing in patient with the gunshot is a dynamic and complex process that requires a suitable approach to promote the healing process. The reason for this is that such wounds are usually deep and extensive. Modern wound dressing in perforated wounds without a fracture or neurovascular injury may be considered for wound healing of outpatients. We report on an 18-year-old girl patient with gunshot wound who was referred to the wound clinic. The symptoms of wound dehiscence in the periumbilical site were observed following to lose suture after primary surgery. Therefore, a new special dressing approach was adopted. Healing of periumbilical wound was successfully achieved at the end.

## Introduction

Gunshot wound leads to deep soft tissue damage knowing as penetrating trauma. Caregivers are usually confronted with the challenges associated with delayed and impaired wound healing [1]. In a patient with the gunshot, wound healing is a dynamic and complex process that requires a suitable approach to promote the healing process because the wound is usually deep and extensive. Debridement, sufficient fasciotomy, complete drainage of the wound and sterile dressing are the treatments for penetrating gunshot wounds [2]. A surgical revision of the wounds is usually seen for all penetrating gunshot injuries; however, modern wound dressing in perforated wounds without a fracture or neurovascular injury may be considered for wound healing of outpatients. Under controlled circumstances, simple perforating wounds have been shown to heal uneventfully with the new approach of dressing changes. Modern wound dressing was the arrival in the 20th century. Two decades later, much has been proven around the benefits of moist dressing it is important to the selection of a material for a particular wound to achieve faster healing [3].

In the absence of evidence-based studies, the recommendations for the treatment of gunshot are based on personal experience. The purpose of this report is to provide a new dressing approach related to a deep gunshot wound in 18 years-old girl patient.

## Case presentation

An 18-year-old girl patient with gunshot wound due to familial upset was referred to the wound clinic affiliated with in September 2021. She had a ruptured small intestine following a gunshot wound and underwent a laparotomy and simultaneously using of Broad-spectrum Antibiotics Vancomycin(1 g/daily), and Ceftazidime (1 g/daily). Seven days after hospitalization, oral feeding was started with considering bowel sounds and then she discharged from trauma medical center to home with some suture on her skin abdomen covered by transparence dressing.

**Fig. 1:**
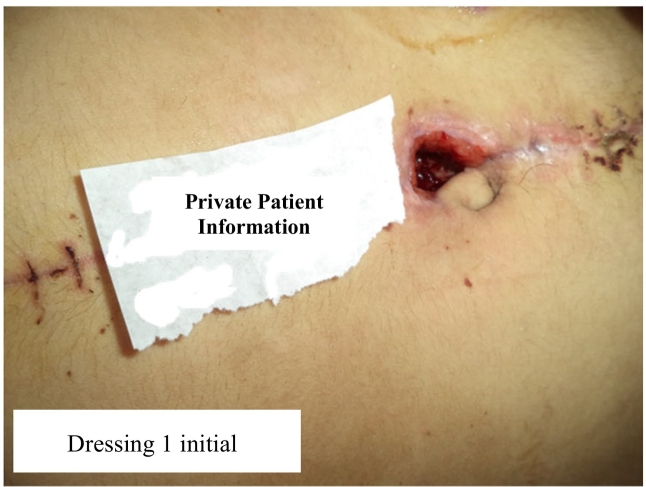
Successful topical healing process of a gunshot wound in 18-year-old girl.

One week after discharge from hospital, she was readmitted to the wound clinic with opened sutures about 2 inch a wound about 2 inch in diameter in periumbilical site of her abdomen, possibly due to inadequate care. Other physical conditions were normal. Vital signs were stable. There were no abnormal findings in laboratory evaluation. Coagulation studies were normal; which was very important to us. Considering recent open surgery, risk of infection, and surgical cost, non-invasive dressing intervention was recommended. In initial physical examination the symptoms of wound dehiscence and necrosis particles in the periumbilical site were observed after loosening the suture (Fig. 1).

**Fig. 2:**
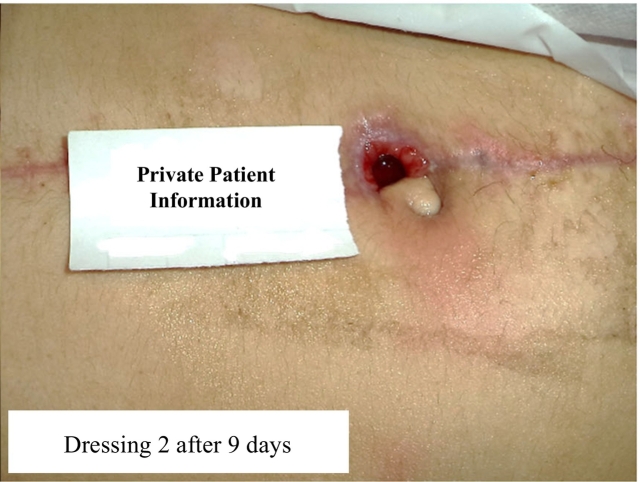
Successful topical healing process of a gunshot wound in 18-year-old girl.

As a suitable treatment option, a new special dressing approach was followed by an expert nurse skilled in the healing of wound and ostomies. At first, the wound was cleaned using normal saline serum and Nivasha spray (including Silver Nanoparticles), and then, dressed using Hydrocolloid gel for debridement every 48 h for three times. After 6 days, the wound was clear of the necrosis and free from proliferation; however, the unpleasant odor-free secretions of the wound grew. At the end of this step, the size of the wound became 1.8 inch wide (Fig. 2).

**Fig. 3:**
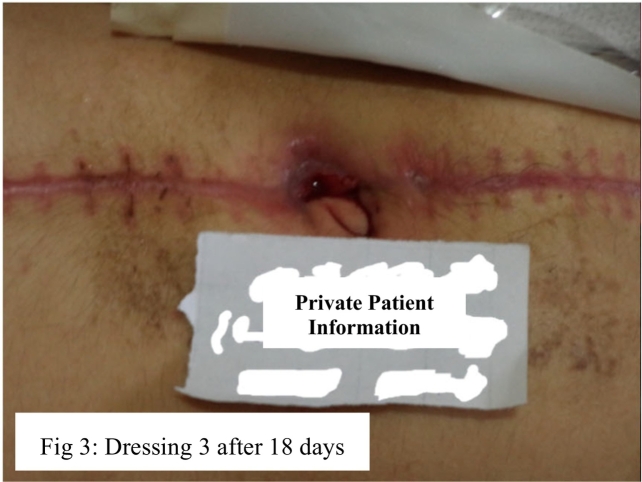
Successful topical healing process of a gunshot wound in 18-year-old girl.

In the second step, since the wound was exposed, Tender-Wet 24 dressing every 72 h for 3 times was used. Tender-Wet 24 dressing includes ringer for scrubbing, discharge absorbent foam and Polyhexamethylene Biguanide (PHMB) that is a polymer used as a disinfectant and antiseptic. During this period, the size of the wound gradually became 0.8 in. in diameter and the secretions of the wound declined. The wound was reddish and exudative; however, there was no evidence of infection. Thus, the wound was washed by normal saline serum to exudate and trap the bacteria. The Aquacell hydro-fiber with Ag extra was used every four days for twice until contracts all sides of the wound. In the end, the size of the wound became 0.5 in. in diameter and the wound secretions declined (Fig. 3).

**Fig. 4:**
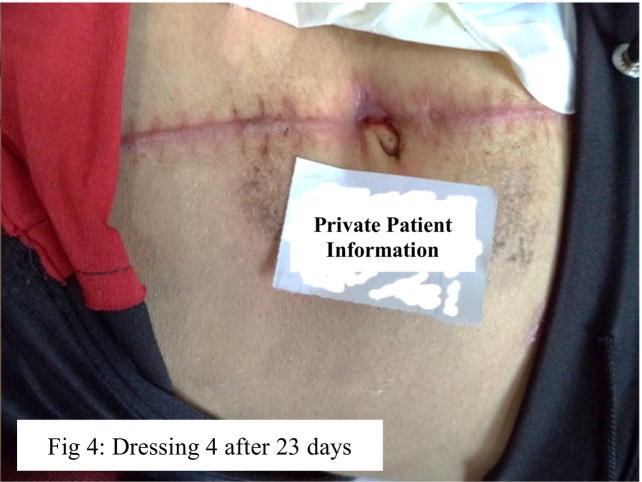
Successful topical healing process of a gunshot wound in 18-year-old girl.

Afterwards, ColActive Plus Ag extra dressing was used every three days twice to create a moist environment for tissue formation and epithelialization. The ColActive Plus Ag extra includes collagen and surfactant for removing biofilms. After this dressing process, the size of the wound became 0.12 in. in diameter and to protect the newly formed tissue, non-adhesive foam dressing was used (Fig. 4). Eventually, the wound was healed and inflammation-free skin was formed without disruption and an unpleasant odor. (Appendix 1).

## Discussion

Hydrocolloid dressing, as one of the advanced wound dressings, is extensively applied. It is a closed dressing, which can preserve the release of biologically active substances and form low oxygen tension on the surface and stimulate the release of interleukins. Therefore, it can enhance the bactericidal effect locally and repair and protect the damaged skin.

Hydrocolloid dressings have active components such as sodium carboxymethyl cellulose particles, medical tape and calcium alginate, and its absorptive dressing [4]. Hydrocolloid dressings, which are widely available, are produced by different companies. It is made of a combination of gel forming agents with elastomers and adhesives. Not only it is permeable to water and vapor, it is also impermeable to bacteria. Therefore, Hydrocolloid dressings have the properties of debridement, absorb wound exudates, and expedite the healing process [5].

It this case, soft tissue debridement was performed by hydrocolloid gels every 48 h for three times using normal saline serum and Nivasha spray at the first stage. Dhivya, Padma, & Santhin Recommended hydrocolloid dressings to remove necrosis [6]. In addition, a study showed that healthy granulation layer formed after applying hydrocolloid dressing to the wound [7], [8].

In addition, TenderWet 24 is one of the specific types of Moisture-activated polyacrylate wound dressing- multi-layered with a superabsorbent polyacrylate core that may enhance selective autolytic debridement. This dressing is rich in sodium, chloride, calcium, and potassium ions. The core of TenderWet 24 has a high affinity of protein, therefore, it absorbs wound exudate and bacterial toxins from the wound surface. TenderWet 24 as an anti-bacterial absorbent pad was used every 72 h for three times. Mancini et al. reported that activated TenderWet 24 is applied for debridement of all type of wounds and at the same time it is able to handle microorganisms and reduce the number of viable germs. [9].

Aquacel Hydrofiber dressing is a new cost-effectiveness and moisture-retention dressing that consists of soft non-woven sodium carboxymethylcellulose fibers and forms a gel on contact with wound fluid. The dressing promotes a moist wound-healing environment and retains wound exudates by vertical absorption. The fibrin is collected between the dressing and wound surface and provides adherence of the dressing to the wound without ingrowth of tissue into the dressing. Therefore, the ease of application and removal also reduces pain at dressing change. Therefore, it is beneficial for both patient and caregiver. [10]. Aquacel Hydrofiber was used with Ag extra dressing twice until contracts at all sides of the wound. Ghumman and Malic used Aquacel Hydrofiber with Ag extra dressing for epithelialization of complicated burns wounds in pediatrics[11]. Nangole found that treatment with Aquacel Ag silver Hydrofiber dressings can decrease mortality in patients with Toxic epidermal necrolysis [12]. Huang et al. showed that Aquacel Ag with Vaseline gauze decreased pain and labor costs [13].

In the next step, ColActive Plus Ag extra dressing including collagen and surfactant was used for removing biofilms. ColActive Plus Ag extra-dressing is a common procedure for covering superficial wounds. It covers the exposed free nerve endings in the dermis; thereby, reducing pain and infection. The Skin graft is one of the common causes of using ColActive Plus Ag extra dressing. After harvesting the split skin graft, dermis is exposed at the donor site, commonly resulting in the reduction in postoperative pain[14]. Mehta et al. showed that, compared with conventional dressing, second-degree burn wounds are well-treated with ColActive Plus Ag extra dressing in the form of good healing, control of infection, and reduction of pain without any serious complications [15].

Finally, we used Biatain non-adhesive foam dressing. Biatain is a soft hydrophilic foam dressing with an elastic semipermeable film backing that impairs bacterial colonization. It has no wound-contact layer. The absence of a contact layer can allow the dressing to adhere to the wound and granulation tissue to grow into the foam matrix. Therefore, Biatain dressing is very efficient. It has excellent exudate management that leads to a rapid initiation of the healing process [16]. This dressing sequence was used to repair the wound in our patient and achieve all targets of wound healing (debridement, scrubbing, moisturizing, exudation, relieving pain and repairing) at the same time.

## Conclusion

Healing of gunshot wound in the periumbilical site was successfully achieved at the end of change dressing sessions. The literature review shows that there is no similar study on the specific dressing sequence used here. Therefore, there is a need for doing more study in the future and in different wound healing. The new approach of wound dressing for gunshot wound is also recommended.

## Ethical approval

As a case report, ethical committee approval was waived. Written consent from the patient was obtained to use the photo and case information for professional purposes.

## Declaration of competing interest

All the authors disclose any financial and personal relationships with other people or organizations that could inappropriately influence (bias) the contents of this paper.

## Figures and Tables

**Figure:**
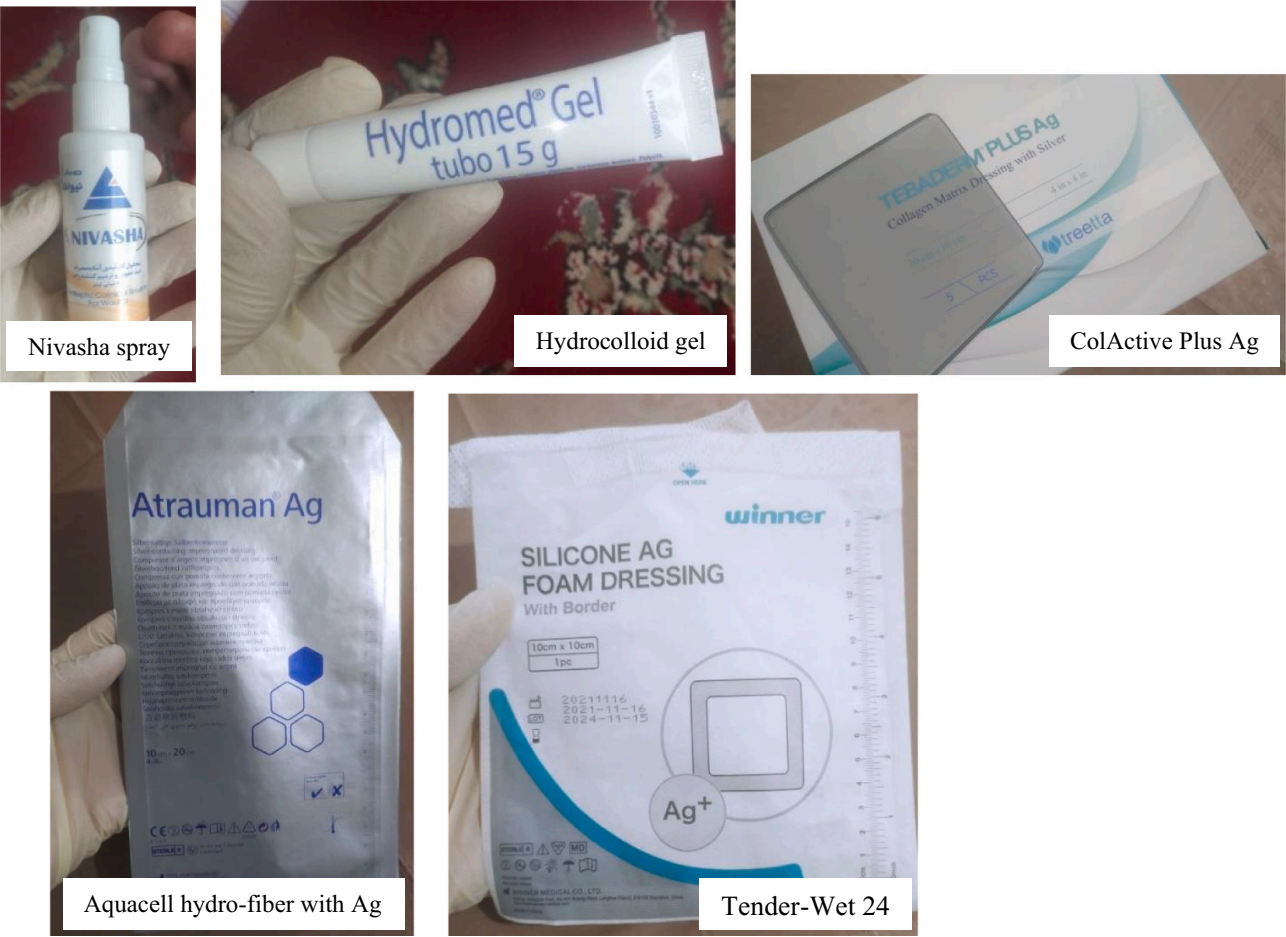


## References

[1] Melamed E., Blumenfeld A., Kalmovich B., Kosashvili Y., Lin G., Korngreen A., Mirowsky I., Mosheiff R., Robinson D., Salai M. (2007). Prehospital care of orthopedic injuries. Prehosp.Disaster Med..

[2] Bekdache O., Paradis T., Bracco D., Elbahrawy A., Khwaja K., Deckelbaum D.L., Fata P., Beckett A., Razek T., Grushka J. (2019). Intermittent use of resuscitative endovascular balloon occlusion of the aorta in penetrating gunshot wound of the lower extremity. Can. J. Surg..

[3] Shah J.B. (2011). The history of wound care. J.Am.Coll.Certif.Wound Spec..

[4] Sood A., Granick M.S., Tomaselli N.L. (2014). Wound dressings and comparative effectiveness data. Adv.Wound Care.

[5] Sadati L., Froozesh R., Beyrami A., Khaneghah Z.N., Elahi S.A., Asl M.F., Salehi T. (2019). A comparison of three dressing methods for pilonidal sinus surgery wound healing. Adv. Skin Wound Care.

[6] Bishopp A., Oakes A., Antoine-Pitterson P., Chakraborty B., Comer D., Mukherjee R. (2019). The preventative effect of hydrocolloid dressings on nasal bridge pressure ulceration in acute non-invasive ventilation. Ulster Med.J..

[7] Cuschieri L., Debosz J., Miiller P., Celis M. (2013). Autolytic debridement of a large, necrotic, fully occluded foot ulcer using a hydrocolloid dressing in a diabetic patient. Adv. Skin Wound Care.

[8] Das S., Baker A.B. (2016). Biomaterials and nanotherapeutics for enhancing skin wound healing [Review]. Front.Bioeng.Biotechnol..

[9] Mancini S., Cuomo R., Poggialini M., D'Aniello C., Botta G. (2018). Autolytic debridement and management of bacterial load with an occlusive hydroactive deressing impregnated with polyhexamethylene biguanide. Acta Biomed..

[10] Cohn S., Lopez P., Brown M., Namlas N., Jackowski J., Li P., Mishkin D., Lopez J. (2004). Open surgical wounds: how does aquacel compare with wet-to-dry gauze?. J. Wound Care.

[11] Ghumman A., Malic C. (2019). A novel application for aquacel ag in paediatric frostbite. Burns Open.

[12] Nangole F. (2018). Silver hydrofiber dressings in the management of patients with toxic epidermal necrolysis: a case series. Wound Heal. South. Afr..

[13] Huang S.H., Lin C.H., Chang K.P., Wu S.H., Lin S.D., Lai C.S., Ou S.F., Lee S.S. (2014). Clinical evaluation comparing the efficacy of aquacel Ag with vaseline gauze versus 1% silver sulfadiazine cream in toxic epidermal necrolysis. Adv. Skin Wound Care.

[14] Yen Y.H., Lin C.M., Hsu H., Chen Y.C., Chen Y.W., Li W.Y., Hsieh C.N., Huang C.C. (2018). Skin graft fixation using hydrofiber (Aquacel(R) Extra). Ann. Plast. Surg..

[15] Mehta M.A., Shah S., Ranjan V., Sarwade P., Philipose A. (2019). Comparative study of silver-sulfadiazine-impregnated collagen dressing versus conventional burn dressings in second-degree burns. J.Fam.Med.Prim.Care.

[16] Walker R.M., Gillespie B.M., Thalib L., Higgins N.S., Whitty J.A. (2017). Foam dressings for treating pressure ulcers. Cochrane Database Syst. Rev..

